# Inter-unit variability in two ParvoMedics TrueOne 2400 automated metabolic gas analysis systems

**DOI:** 10.1007/s00421-012-2483-9

**Published:** 2012-09-04

**Authors:** D. J. Macfarlane, H. L. Wu

**Affiliations:** Institute of Human Performance, The University of Hong Kong, Pokfulam, Hong Kong

**Keywords:** Variation, Agreement, Technological error, ParvoMedics, Gas analysis

## Abstract

Knowing the inter-unit variability, especially the technological error, is important when using many physiological measurement systems, yet no such inter-unit analysis has been undertaken on duplicate automated gas analysis systems. This study investigated the inter-unit performance of two identical ParvoMedics TrueOne 2400 automated gas analysis systems during a range of submaximal steady-state exercises performed on an electromagnetic cycle ergometer. Fifteen adult males were tested on two separate days a rest, 30, 60, 90, and 120 Watts with the duplicate gas analysis units arranged (1) collaterally (2 min of steady-state expired gas was alternately passed through each system), and (2) simultaneously (identical steady-state expired gas was passed simultaneously through both systems). Total within-subject variation (biological + technological) was determined from the collateral tests, but the unique inter-unit variability (technological error between identical systems) was shown by the simultaneous tests. Absolute percentage errors (APE), coefficient of variations (CV), effect sizes and Bland–Altman analyses were undertaken on the metabolic data, including expired ventilation (*V*_E_), oxygen consumption (*V*O_2_) and carbon dioxide production (*V*CO_2_). The few statistically significant differences detected between the two duplicate systems were determined to have small or trivial effect sizes, and their magnitudes to be of little physiological importance. The total within-subject variations for *V*O_2_, *V*CO_2_ and *V*_E_ each equated to a mean CV and mean APE value of ~4 and ~6 %, whilst the respective inter-unit technological errors equated to ~1.5 and ~2.1 %. The two ParvoMedics TrueOne 2400 systems demonstrated excellent inter-unit agreement.

## Introduction

The research community often benefits by being able to compare physiological data across studies providing that non-identical devices produce the same outputs when tested under the same conditions (good inter-system agreement); for example, when several automated gas analysis systems from different manufacturers provide comparable metabolic data on the same subjects (Cooper et al. 2009). Yet the opposite is quite common, with non-identical gas analysis systems producing less than acceptable agreement (Miles et al. 1994).

When multiple identical devices are used to collect and compare data from several measurement localities (e.g., each site having the same model gas analysis system), it is essential to test that each unit of the systems is not significantly biased and indeed produces comparable results (i.e., low inter-unit variability). Assessing inter-unit variability is quite different from how the more common inter-system accuracy (validity) is traditionally measured. Inter-unit variability is a measure of how reproducible the results are when two or more identical models of the measurement device are tested under the exact same situations, including conditions that mimic the future research methodology, and all units are tested at the same time. In comparison, inter-system accuracy (validity), especially for gas analysis systems, typically compares only one model of an instrument against a quite different criterion/gold device, typically a Douglas Bag system (Douglas 1911). Ideally both metabolic measurements should occur “simultaneously” during the same steady-state activity (e.g., (Crouter et al. 2006), although they are often taken at different times during steady-state activity due to interference between the measurement systems (i.e., “collaterally” e.g., Vogler et al. 2010).

The total within-subject variance, as measured between two gas analysis systems on the same subject(s) over two different time periods, is the sum of the within-subject biological (temporal) variation + technological error variation. When comparison between two, or more, identical systems occurs over exactly the same time periods and same conditions (inter-unit agreement), the biological error component is removed, allowing the true technological error between the duplicate units to be measured. Knowing the technological variability between duplicate units is important when collating data across multi-centre trials each with a single unit, or within-laboratory trials using multiple units.

A number of studies have examined the variance in metabolic gas analysis systems. Using a Krogh-type gasometer, Taylor (1944) reported the CV of the total variance for submaximal *V*O_2_ (6.5 %) and *V*_E_ (8.0 %), plus maximal values: *V*O_2max_ (7.5 %) and *V*_Emax_ (7.2 %), and estimated *V*O_2_ “method error” (technological error) to be ~0.1–0.3 %. Using Douglas bags, Taylor et al. (1955) reported the total variability in repeated *V*O_2max_ tests to be 2.4 %, lower than the 5.5 % reported by Mitchell et al. (1958), but much higher than the 0.26 % of McArdle et al. (1973). Whilst the novel intra- and inter-lab reliability data of Jones and Kane (1979) showed total percent variations in *V*O_2_, *V*CO_2_, and *V*_E_ of 3.8, 4.2 and 8.0 %, respectively, for their short-term intra-lab tests. Using 80 repeated *V*O_2max_ trials with Douglas bags, Katch et al. (1982) reported the total within-subject variation to be 5.6 %, with biological error accounting for ~90 % of this: the technological error was therefore ~10, or <0.4 % of the total *V*O_2max_ variance and almost identical to the findings from Armstrong and Costill’s (1985) Rayfield system. In both these studies, the low technological error reflected intra-unit reliability and is likely to be lower than the unknown inter-unit variability. More recently James and Doust (1997) reported a Douglas bag technological error CV of 1.4 % using a same subject/same(within)-bout protocol, but a higher CV of 2.1 % for repeated *V*O_2_ measurement on the same subject, but on a different exercise bout (biological and technological error).

Yet no study has yet reported an inter-unit variability study on any automated gas analysis system and it is unknown how much technological error occurs due purely to calibration and/or hardware differences between two or more identical units of the same system. Although two studies have reported data collected from multiple models of the same automated gas analysis system using the same subjects across each device. Yule et al. (1996) measured eight subjects during two submaximal and one maximal test at the same time of day at three separate laboratories, all using similar Sensormedics 2900 metabolic carts. Their results showed significant differences in both *V*O_2max_ and respiratory exchange ratio (RER) between labs, with the mean *V*O_2max_ varying by 4–14 % between the three labs. The HERITAGE Family study used eight travelling subjects to examine the reproducibility of physiological variables during two submaximal and one maximal test across four laboratories, also using similar Sensormedics 2900 metabolic carts (Gagnon et al. 1996; Skinner et al. 1999; Wilmore et al. 1998). The submaximal tests produced a coefficient of variation (CV) for *V*O_2_, *V*CO_2_ and *V*_E_ that ranged from 3.5 to 4.7 %, 3.8 to 5.8 % and 5.1 to 6.9 %, respectively; whilst the maximal test produced respective CV values of 4.1, 5.5 and 8.5 %. Although both studies reported insightful data on the reproducibility between labs using the same measurement systems, neither study was specifically designed to reveal the true technological error between identical units. This type of inter-unit variability study would have required both studies to use absolute duplicates of the same metabolic analysis systems (identical age, identical maintenance, identical volume and gas calibrations, and identical testing conditions). The variations reported in these two studies between the Sensormedics 2900 machines will therefore have been inflated by an unknown mixture of these technological sources of contamination; moreover, the results are likely to have contained a measureable biological variation as the subjects were tested on different days. Another study has reported metabolic data from two early ParvoMedics TrueMax systems configured to measure simultaneously gas exchange using the same eight subjects (Bassett et al. 2001). However, these two systems were also not identical, as the study aimed to compare the validity of the inspiratory system against the expiratory system, thus the technological error between two identical gas analysis systems remains unknown.

**Fig. 1:**
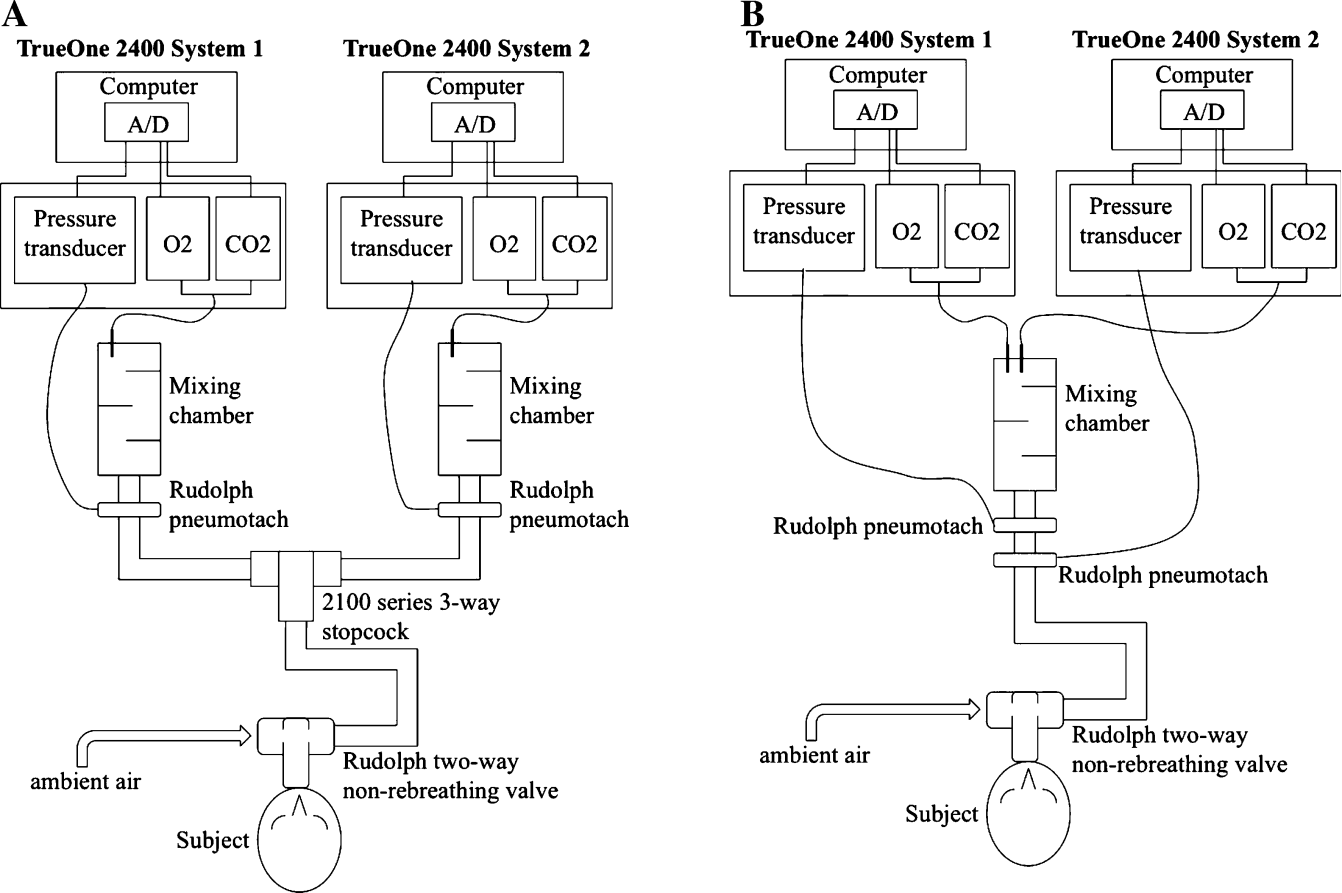
Illustration of the two experimental set-ups. **a** Collateral configuration with alternating measurements of the same steady-state activity made by the two identical gas analysis units. **b** Simultaneous configuration with simultaneous measurements of the same steady-state activity made by both identical gas analysis units, but sharing a common mixing chamber

A recent review on the performance of gas analysis systems (Atkinson et al. 2005) has also stressed the importance of detailing the sources of variation in gas analysis systems. The aim of this study was therefore to examine the variability of two identical ParvoMedics TrueOne 2400 automated gas analysis units during a range of submaximal bouts of exercise, by measuring: (1) the combined biological and technological errors, where the steady-state expired gases were alternately directed into each system (set-up for “collateral” measurement of non-identical gases/times: see Fig. 1a); and (2) technological error only, where the expired gases flowed continuously through both systems (set-up for “simultaneous” measurement of identical gases/times: see Fig. 1b).

## Methods

### Participants

A sample of fifteen apparently healthy Hong Kong Chinese male volunteers were recruited with the following characteristics (mean ± SD): age 21.7 ± 1.4 year; height 175.1 ± 6.6 cm; mass 74.0 ± 11.4 kg. All subjects provided written informed consent, after the project was approved by the Research Ethics Committee of the University of Hong Kong.

### Equipment

ParvoMedics TrueOne 2400 systems. Both TrueOne 2400 units (see http://www.parvo.com) were identical models of an automated “mixing chamber” metabolic gas analysis system, comprising a measurement module connected to a Dell Optiplex 790 computer running Windows 7 and ParoMedics OUSW 4.3.4 (v.20111228) data acquisition/analysis software. The two 2400 units were of identical age, with the measurement modules having sequential manufacturing serial numbers (3845HU-NL, 3846HU-NL); each unit had been installed by a ParvoMedics’ factory staff within the previous 3 months and had been used ~10 times each. Expired gas passed sequentially through a Hans Rudolph (Shawnee, KS) 2700 two-way non-rebreathing valve, a saliva collector trap, a Creative Biomedics Inc (San Clemente, CA, USA) CB-1501-2 filter, a Hans Rudolph series-3813 heated pneumotachometer, and into a 4 L mixing chamber. The mixed expired gas was then continuously sampled using a 61 cm Nafion tube (Permapure, Toms River, NJ, USA) by a paramagnetic oxygen analyzer (0–25 % range with 0.1 % accuracy) and an infrared carbon dioxide analyzer (0–10 % range, with 0.1 % accuracy) to measure fractions of expired oxygen (*F*_EO2_) and carbon dioxide (*F*_ECO2_). Both systems were turned-on at least 30 min prior to testing and calibrated according to the manufacturer’s instructions. This involved the pneumotachometer being calibrated using five strokes of a 3L syringe (Hans Rudolph series-5530) at graduated flow-rates from 50 to 80 L min^−1^ up to >400 L min^−1^. The gas analyzers were calibrated using a two-point fully automated process involving room air and a certified gas (3.98 % CO_2_, 16.03 % O_2_, balance N_2_: Airgas Specialist Gases, Lenexa, KS). The certified calibration gas cylinders for each system came from the same production run (Lot ZP12Y161BB) and used identical Victor CGA973 pressure regulators (Denton, TX). A minimum of two flowmeter and two gas analyzer calibrations were performed before each test. The ambient temperature, barometric pressure and relative humidity were entered into each computer from each system’s corresponding weather station (model WS-2080; Ambient Weather, Chandler, AZ, USA); each weather station had been corrected and matched with the ParvoMedics’ factory standards.

Each participant sat on a Lode (Groningen, The Netherlands) Corival 400 electromagnetically braked cycle ergometer, wore a noseclip, and breathed through a mouthpiece attached to a Hans Rudolph 2700-series non-rebreathing valve that was held by a Hans Rudolph 2726 head-support. Each participant also wore a telemetric heart rate monitor (Polar, Kempele, Finland). The experimental set-up differed slightly between the two tests.

#### Collateral set-up (Fig. 1a)

Expired gas from the 2700 valve passed through a 13-cm corrugated plastic tube (35 mm diameter) to a Hans Rudolph 2100-series 3-way stopcock, so the experimenter could alternately direct it into one of the two TrueOne 2400 units via a 180-cm corrugated plastic tube (35 mm diameter). The flowmeters for each system were calibrated manually within ~1 min of each other, whilst the automated gas calibrations were done simultaneously before each test. Each participant underwent a continuous incremental submaximal exercise test using five steady-state periods (Rest, 30, 60, 90, 120 W at constant pedal frequency of 60 rpm), each lasting 7 min. A steady-state was achieved over the first 3 min, then 2 min of steady-state expired gas was directed into one 2400 unit (3rd–5th minute), then switched to the alternate 2400 unit for the 5th–7th minute; after the 7th minute, the work rate was increased and the sequence was repeated. During the initial 3 min of each exercise stage, the 2100 stopcock was used to periodically alternate gas into each system to ensure each mixing chamber had similar mixed expired gas compositions. To prevent an order effect, the sequence of the 2400 units was balanced by switching them after each participant.

#### Simultaneous set-up (Fig. 1b)

Expired gas from the 2700 valve passed directly into both the TrueOne 2400 units via a 180-cm corrugated plastic tube (35 mm diameter), with the two pneumotachometers separated by a 13-cm corrugated plastic tube (35 mm diameter). To prevent an order effect, the positioning of the pneumotachometers was balanced by switching them after each participant. Manual calibration of both pneumotachometers was then able to be performed simultaneously before each test, along with simultaneous automated calibrations of both sets of gas analyzers. After a period of at least 1 week from the collateral test, each participant underwent a second continuous incremental submaximal exercise test using the same five steady-state bouts (Rest 30, 60, 90, 120 W), but each lasting only 5 min (the previous 5th–7th minute period was not required, as steady-state gas passed simultaneously through both units over the 3rd–5th minute), before the work rate was increased. To ensure exact ventilatory synchrony between the two metabolic systems, participants were asked to momentarily hold their breath (i.e., zero gas flow), whilst data acquisition for both units was simultaneously started, thereby ensuring each unit recorded an identical number and sequence of breaths.

#### Data processing/analysis

Both units reported a 30 s average in all metabolic data. At the end of each test, the data file was exported and analyzed using MicroSoft Excel 2011, with the mean of the final 90 s of each 2 min period of steady-state used in the final analysis. We selected a variety of reliability statistics for each variable, as has been recommended (Atkinson and Nevill 1998; Hodges et al. 2005). These included: the absolute percentage error (APE) and individual coefficient of variation expressed as a percentage of the mean score (CV) (McClain et al. 2007); the effect size (ES) being the magnitude of the group difference relative to the overall standard deviation (Cohen 1988); Bland–Altman plots (Bland and Altman 1986); and shifts in mean scores using paired *t* tests with Holm’s sequential Bonferroni adjustments (Holm 1979).

## Results

**Fig. 2:**
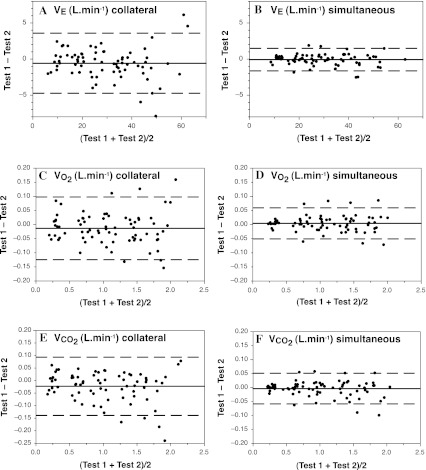
Bland–Altman plots from the collateral (**a**, **c**, **e**) and simultaneous (**b**, **d**, **f**) tests, showing the error scores for the two gas analysis systems (Test unit 1 − Test unit 2); data shown for *V*_E_ (**a**, **b**), *V*O_2_ (**c**, **d**), and *V*CO_2_ (**e**, **f**). *Solid horizontal line* indicates the mean error, whilst the *dashed horizontal lines* indicate the 95 % LOA

Table 1 shows that the metabolic data from the two automated gas analysis units arranged collaterally were very similar across all workloads, with no statistically significant differences in any of the mean comparisons (after Holm–Bonferroni adjustment). Except for the Rest condition (when the relative error was expected to be higher), the APE and CV values tended to range between 2 and 4 %. All effect sizes were below 0.32, with 21 of the 30 (70 %) classified (Saunders 2004) as being trivial (<0.2) and the remaining 30 % as small (0.2–0.5). The Bland–Altman plots in Fig. 2a, c, e, show very minor systematic error (bias) between the two collateral systems, with almost no proportional random error, and small 95 % limits of agreement (LOA).

**Table 1:** Physiological responses from the graded exercise test using two “collateral” systems (1 and 2: mean ± SD), and *p* value from paired *t* tests, absolute percentage error (APE), coefficient of variation (CV), and effect size

Variable	System 1	System 2	Paired *t* *p* value	APE mean (range)	CV mean (range)	Effect size
*V*_E_ (L min^−1^)						
Rest	11.91 ± 6.29	11.84 ± 6.14	0.850	12.4 (2.0–39.7)	8.4 (1.4–23.4)	0.01
30 W	19.57 ± 4.29	20.01 ± 4.47	0.110	4.3 (0.5–12.9)	2.9 (0.4–8.6)	0.11
60 W	26.83 ± 3.40	27.41 ± 3.50	0.280	6.7 (0.0–18.1)	4.6 (0.0–11.7)	0.17
90 W	37.37 ± 4.49	37.94 ± 3.43	0.160	3.1 (0.1–11.7)	2.2 (0.1–7.8)	0.15
120 W	49.33 ± 7.49	50.71 ± 5.64	0.170	6.2 (0.4–17.3)	4.2 (0.3–11.2)	0.21
*V*O_2_ (L min^−1^)						
Rest	0.30 ± 0.06	0.31 ± 0.06	0.780	11.6 (1.1–26.2)	8.1 (0.8–20.2)	0.05
30 W	0.73 ± 0.08	0.74 ± 0.08	0.200	4.7 (0.0–16.5)	3.2 (0.0–10.8)	0.18
60 W	1.06 ± 0.08	1.07 ± 0.08	0.380	4.2 (0.7–9.4)	2.9 (0.5–7.0)	0.17
90 W	1.44 ± 0.09	1.46 ± 0.07	0.340	3.1 (0.1–10.5)	2.2 (0.1–7.1)	0.20
120 W	1.83 ± 0.14	1.85 ± 0.10	0.320	3.7 (0.2–8.5)	2.6 (0.1–5.7)	0.18
*V*CO_2_ (L min^−1^)						
Rest	0.28 ± 0.09	0.28 ± 0.10	0.720	12.6 (1.0–37.4)	8.6 (0.7–22.3)	0.04
30 W	0.63 ± 0.08	0.64 ± 0.09	0.160	4.5 (0.5–14.9)	3.1 (0.4–9.8)	0.17
60 W	0.95 ± 0.08	0.97 ± 0.08	0.120	5.2 (0.1–13.5)	3.5 (0.1–9.0)	0.32
90 W	1.36 ± 0.11	1.39 ± 0.09	0.090	3.3 (0.2–14.0)	2.2 (0.2–9.2)	0.27
120 W	1.77 ± 0.19	1.82 ± 0.17	0.070	3.9 (0.1–13.4)	2.7 (0.1–8.9)	0.26
*F*_EO2_ (%)						
Rest	17.58 ± 0.60	17.57 ± 0.55	0.890	1.1 (0.2–2.9)	0.8 (0.1–2.0)	0.02
30 W	16.42 ± 0.54	16.43 ± 0.58	0.810	0.6 (0.1–1.9)	0.4 (0.1–1.3)	0.02
60 W	16.14 ± 0.45	16.18 ± 0.43	0.500	0.9 (0.0–2.8)	0.6 (0.0–2.0)	0.08
90 W	16.21 ± 0.37	16.24 ± 0.31	0.600	0.8 (0.0–2.5)	0.6 (0.0–1.7)	0.08
120 W	16.37 ± 0.37	16.44 ± 0.36	0.170	1.1 (0.2–2.1)	0.8 (0.1–1.5)	0.20
*F*_ECO2_ (%)						
Rest	3.06 ± 0.40	3.06 ± 0.37	0.840	3.7 (0.1–12.6)	2.6 (0.0–8.4)	0.02
30 W	4.03 ± 0.46	4.02 ± 0.47	0.860	2.2 (0.4–7.6)	1.6 (0.3–5.6)	0.01
60 W	4.41 ± 0.42	4.42 ± 0.43	0.560	2.1 (0.1–5.4)	1.5 (0.1–3.7)	0.05
90 W	4.55 ± 0.38	4.55 ± 0.33	0.890	2.6 (0.6–7.9)	1.8 (0.4–5.4)	0.01
120 W	4.48 ± 0.32	4.46 ± 0.34	0.610	3.1 (0.4–6.6)	2.2 (0.3–4.5)	0.07
RER						
Rest	0.90 ± 0.13	0.90 ± 0.15	0.930	4.1 (0.0–14.3)	2.9 (0.0–10.9)	0.01
30 W	0.86 ± 0.04	0.86 ± 0.06	0.770	2.6 (0.1–7.5)	1.8 (0.0–5.1)	0.05
60 W	0.89 ± 0.04	0.90 ± 0.04	0.090	2.5 (0.2–7.6)	1.7 (0.1–5.2)	0.29
90 W	0.95 ± 0.03	0.95 ± 0.05	0.210	2.0 (0.0–7.3)	1.4 (0.0–5.0)	0.22
120 W	0.97 ± 0.04	0.98 ± 0.05	0.070	2.6 (0.3–4.9)	1.8 (0.2–3.4)	0.30

* Significantly different means by paired *t* test with Holm’s sequential Bonferroni adjustment

The metabolic data in Table 2 from the two gas analysis units arranged simultaneously show that the inter-unit agreement was higher compared to the collateral test, as predicted. Even though the *p* values from the paired *t* tests for *F*_EO2_ and RER were often very small (and traditionally regarded as being significant, *p* < 0.05), this reflected the extremely consistent intra-unit variation (very low standard deviations or ‘noise’) rather than a relatively large bias in the inter-unit signal. As a result, even a very small (and physiologically trivial) systematic error/bias between the two units would appear to be large relative to the small standard deviation. Yet only one pairing produced a statistically significant difference between mean values (RER at 120 W) after Holm–Bonferonni adjustment; but the small APE and CV for this pair of 0.8 and 0.6 %, respectively, and a trivial effect size (0.16) all confirmed this was a physiologically insignificant difference. Overall, most APE and CV values for the simultaneous comparison were very small, typically varying around 0.5–2.0 %, with all effect sizes being trivial (maximum of 0.16); indeed, 77 % of the effect sizes were <0.1. This extremely good inter-unit agreement was further confirmed by the Bland–Altman plots B, D, and F in Fig. 2, that show almost no systematic error, together with uniform random error, and very small 95 % LOA.

**Table 2:** Physiological responses from the graded exercise test using two “simultaneous” systems (1 and 2: mean ± SD), and *p* value from paired *t* tests, absolute percentage error (APE), coefficient of variation (CV), and effect size

Variable	System 1	System 2	Paired *t* *p* value	APE mean (range)	CV mean (range)	Effect size
*V*_E_ (L min^−1^)						
Rest	12.26 ± 3.26	12.21 ± 3.30	0.555	2.6 (0.1–9.2)	1.8 (0.0–6.2)	0.02
30 W	19.70 ± 2.49	19.63 ± 1.94	0.752	2.6 (0.1–10.8)	1.9 (0.1–7.3)	0.03
60 W	27.16 ± 2.75	27.15 ± 2.38	0.991	2.1 (0.2–6.4)	1.5 (0.2–4.4)	0.00
90 W	37.13 ± 5.08	37.19 ± 4.93	0.750	1.7 (0.1–4.1)	1.2 (0.1–2.8)	0.01
120 W	47.86 ± 6.09	48.27 ± 5.73	0.173	1.8 (0.1–6.1)	1.2 (0.1–4.2)	0.07
*V*O_2_ (L min^−1^)						
Rest	0.32 ± 0.05	0.31 ± 0.04	0.102	2.9 (0.9–7.0)	2.0 (0.6–4.8)	0.10
30 W	0.73 ± 0.08	0.73 ± 0.07	0.420	2.6 (0.1–9.2)	1.8 (0.1–6.8)	0.09
60 W	1.05 ± 0.09	1.04 ± 0.08	0.423	1.9 (0.1–7.2)	1.4 (0.1–5.3)	0.08
90 W	1.42 ± 0.08	1.41 ± 0.07	0.327	1.6 (0.1–5.2)	1.1 (0.1–3.8)	0.10
120 W	1.78 ± 0.13	1.78 ± 0.12	0.823	1.6 (0.1–4.5)	1.1 (0.0–3.3)	0.02
*V*CO_2_ (L min^−1^)						
Rest	0.29 ± 0.06	0.29 ± 0.06	0.702	2.4 (0.3–9.2)	1.7 (0.2–6.2)	0.02
30 W	0.64 ± 0.07	0.64 ± 0.06	0.885	2.6 (0.3–10.7)	1.8 (0.2–7.2)	0.01
60 W	0.95 ± 0.07	0.95 ± 0.07	0.856	2.1 (0.2–6.1)	1.5 (0.1–4.5)	0.02
90 W	1.37 ± 0.11	1.37 ± 0.12	0.698	1.7 (0.0–3.9)	1.2 (0.0–2.7)	0.03
120 W	1.75 ± 0.16	1.77 ± 0.15	0.163	1.7 (0.0–5.9)	1.2 (0.0–4.0)	0.10
*F*_EO2_ (%)						
Rest	17.73 ± 0.57	17.75 ± 0.57	0.008	0.1 (0.0–0.4)	0.1 (0.0–0.3)	0.03
30 W	16.46 ± 0.41	16.48 ± 0.41	0.007	0.2 (0.0–0.6)	0.1 (0.0–0.4)	0.06
60 W	16.27 ± 0.35	16.29 ± 0.34	0.012	0.2 (0.0–0.6)	0.1 (0.0–0.4)	0.07
90 W	16.22 ± 0.45	16.25 ± 0.44	0.006	0.2 (0.0–0.6)	0.1 (0.0–0.4)	0.06
120 W	16.34 ± 0.42	16.37 ± 0.41	0.003	0.2 (0.0–0.6)	0.1 (0.0–0.4)	0.07
*F*_ECO2_ (%)						
Rest	2.99 ± 0.39	3.00 ± 0.39	0.081	0.3 (0.0–1)	0.2 (0.0–0.7)	0.01
30 W	4.05 ± 0.37	4.05 ± 0.37	0.497	0.2 (0.0–0.8)	0.2 (0.0–0.6)	0.01
60 W	4.37 ± 0.37	4.37 ± 0.37	0.491	0.3 (0.0–1.0)	0.2 (0.0–0.7)	0.01
90 W	4.60 ± 0.37	4.60 ± 0.37	0.863	0.3 (0.0–0.7)	0.2 (0.0–0.5)	<0.01
120 W	4.56 ± 0.36	4.56 ± 0.36	0.792	0.3 (0.0–0.8)	0.2 (0.0–0.5)	<0.01
RER						
Rest	0.92 ± 0.12	0.93 ± 0.13	0.010	1.1 (0.1–4.1)	0.8 (0.1–2.8)	0.09
30 W	0.87 ± 0.05	0.88 ± 0.05	0.002	0.8 (0.2–2.1)	0.6 (0.1–1.5)	0.14
60 W	0.91 ± 0.04	0.92 ± 0.04	0.002	0.8 (0.1–2.0)	0.6 (0.1–1.4)	0.15
90 W	0.96 ± 0.06	0.97 ± 0.06	0.003	0.9 (0.2–1.9)	0.6 (0.1–1.3)	0.12
120 W	0.98 ± 0.04	0.99 ± 0.05	0.002*	0.8 (0.0–1.8)	0.6 (0.0–1.3)	0.16

* Significantly different means by paired *t* test with Holm’s sequential Bonferroni adjustment

## Discussion

This is the first study to examine the inter-unit variability of metabolic data between two identical automated gas analysis systems. Analysis of the simultaneous set-up permits the first in situ assessment of the technological error that occurs between two identical automated gas analysis units due to the small variations that cannot be fully eliminated in the calibration process and/or due to variations associated with the data-acquisition/processing hardware (e.g., different inter-unit manufacturing tolerances and inherent intra-unit measurement noise). This study also provided data on the added variation created when a small level of biological error was introduced. This added biological error was due to the non-simultaneous sampling of inherently imperfect steady-state measurements of human respiration (the collateral test), as opposed to using ideal steady-state conditions generated by mechanical metabolic calibration systems (Gore et al. 1997; Vogler et al. 2010).

A review of the variability measured between repeated submaximal or maximal tests using recent automated gas analysis systems is beyond the scope of this paper and aspects have been reported elsewhere (Crouter et al. 2006; Hodges et al. 2005; Macfarlane 2001). However, some relevant comparative data indicate the total variations in reliability measures of *V*O_2_, *V*CO_2_, and *V*_E_ over 2 days using the ParvoMedics 2400 system produced a respective CV of 4.7, 5.7 and 7.3 % (Crouter et al. 2006). In comparison, the respective CV values from the collateral tests in this current study of 3.8, 4.0, and 4.5 % are all predictably lower since the variability measured during two sections of the same steady-state on the same day (collateral test), will be lower than that seen during two separate steady-states measured across two different days (Crouter-study). The current study’s collateral testing should therefore represent some of the smallest possible within-subject variation (biological + technological).

If the technological variability (simultaneous tests) is subtracted from the total within-subject variability (collateral tests), the biological variability remains. When this is done for the mean *V*O_2_, *V*CO_2_, and *V*_E_ data, then the respective biological variation APE values are 3.3, 3.8, and 4.4 %, whilst the CV values are 2.3 , 2.5, and 2.9 %. The mean CV for the *V*O_2_ biological variation in our study of 2.3 % is predictably lower than the 5.2 % reported by Katch et al. (1982), since our study used a same day within-exercise comparison, rather than a between-day comparison used by Katch et al. The biological APE of 2.3 % in our *V*O_2_ data from our highest steady-state exercise is very similar to the 2 % estimated by Withers et al. (2000) for *V*O_2max_ data taken across 5 certified Australian laboratories. However, our novel findings show that even when taking measurements by two identical metabolic systems during the same theoretical “steady-state exercise”, some small biological variation is measurable above the between-system technological variation. This measurable biological variation may have been due to a slight upward drift in the metabolic rate due to the relatively slow kinetic change in core body temperature, as heart rate also showed a small mean APE difference of 3.3 % during the collateral tests (compared to a trivial 0.3 % for the simultaneous tests).

The data in Table 2 indicate that the technological error due to differences in calibration and/or instrumentation noise is consistently less than an APE of 3 % and below a CV of 2 %, with variations in measured *V*O_2_ and *V*CO_2_ predominantly being due to variations in *V*_E_ rather than *F*_EO2_ or *F*_ECO2_. The level of agreement between the identical sets of O_2_ and CO_2_ analyzers when measuring “identical” mixed expired gas (simultaneous) was impressively low at 0.1–0.3 % for both APE and CV, indicating that the auto-gas calibration routine and performance of the ParvoMedics gas analyzers were extremely precise. The mean technological variation between our two identical ParvoMedics systems when measuring *V*O_2_ (APE = 2.1 %; CV = 1.5 %) was also predictably higher than the values reported by four other studies (Armstrong and Costill 1985; James and Doust 1997; Katch et al. 1982; Taylor 1944), as our study compared inter-unit variability (differences between two identical systems), rather than the intra-unit variability (repeatability of a single system), which was reported in the above four studies. Despite this, our technological CV of 1.5 % remains considerably lower that than “much of the CV of 5 %” also attributed to technological error from the 2-day test–retest data described by Shephard et al. (2004).

Our technological error was slightly elevated since each of the two 2400 systems used its own ambient weather station for temperature, pressure and relative humidity (see "Methods"), rather than one common laboratory standard. Although these weather stations were re-calibrated to a ParvoMedics factory standard, very small variations (mean APE) were seen in temperature (1.3 %), pressure (<0.1 %), and relative humidity (2.8 %) between the two systems, which would have influenced the technological error in the calculation of *V*O_2_, *V*CO_2_ and *V*_E_. However, the cumulative effect of these inter-unit variations in ambient variables would cause less then a 0.2 % error in the calculation of the important *V*O_2_ variable (Howley et al. 1995; Withers et al. 2000), and is of little physiological importance. Two other factors may have contributed in a minor way to the “simultaneous condition” technological error: (a) the small 13 cm tubing that connected the two pneumotachometers may have added a small downstream deadspace, and (b) heating of the expirate by the proximal pneumotachometer may have caused a small volume increase that was measured by the distal pneumotachometer. However, we surmise that switching the order of the pneumotachometers after each test should have minimized their net effect.

There remains no universally accepted level of precision (nor accuracy) for the measurement of the important metabolic variable of *V*O_2_ (Macfarlane 2001), and although a technical error of measurement (TEM) target of 3 % for *V*O_2max_ has been recommended by Gore (2000), the coefficient of variation appears to be the more commonly used statistic to report data from reliability/precision test–retests. During high intensity *V*O_2_ tests, a CV of ~4–5 % (total within-subject variability in *V*O_2_) is frequently reported in the literature from intra-unit reliability analyses from separate bouts at the same work rate (Armstrong and Costill 1985; Becque et al. 1993; Bingisser et al. 1997; Carter and Jeukendrup 2002; Clark et al. 2008; Crouter et al. 2006; Froelicher et al. 1974; Howley et al. 1995; James and Doust 1997; Jones 1984; Nordrehaug et al. 1991; Rosdahl et al. 2009; Skinner et al. 1999). Although we did not attempt *V*O_2max_ measures during our inter-unit comparison of the ParvoMedics systems, the CV for *V*O_2_ from our highest steady-state work rate was 2.6 % (APE = 3.7 %), which compares very favorably with the often cited 4–5 % range. These results suggest that under very well controlled test–retest situations (i.e., our collateral tests, that involved minimal biological error due to measurements taken within the same steady-state bout, plus minimal technological error due to duplicate ParvoMedics systems), the total variation between two automated gas analysis systems can provide a CV <3 % at high work rates. Furthermore, our APE of 3.7 % also suggests that the targeted 4–5 % limit between two metabolic systems (Babineau et al. 1999) can be achieved, albeit, under very tightly controlled circumstances.

No other study has examined the technological error between two identical systems (our inter-unit simultaneous test), hence comparative data are not available. However, some comparison can be made with the extremely well controlled intra-unit precision tests undertaken using a highly specialized automated *V*O_2_ calibration system (Gore et al. 1997). When measuring identical respiratory gases from the automated calibrator, the three gas analysis systems measured by Gore’s team produced a mean CV <1 % for *V*O_2_, *V*CO_2_, and V_E_. When compared to these excellent intra-unit measures of precision, the mean CV for *V*O_2_, *V*CO_2_, and *V*_E_ was ~1.5 % from our inter-unit comparison (mean APE ~2.1 %). The slightly higher CVs seen in our in-series study remain impressive, since our data were from an inter-unit comparison collected during “real-life” laboratory conditions using actual human volunteers, rather than the intra-unit comparisons using precisely simulated mechanical respiration of Gore et al. (1997). Our mean inter-unit APE of ~2.1 % is also very similar to the ±2 % recommendation for intra-unit reliability suggested by Hodges et al. (2005), indicating that the low inter-unit variability seen between our duplicate ParvoMedics systems can effectively match the tolerances traditionally recommended for intra-unit variability.

This study has several limitations, including that only two duplicate ParvoMedics systems were analyzed, but we have no reason to expect the low variability seen between our systems would not be indicative of other recent TrueOne 2400 systems. Unlike some studies we also did not use any form of automatic gas exchange calibration systems (Gore et al. 1997; Macfarlane and Wong 2011) to test these systems, as we wished to report their performance under “real-life” laboratory conditions, as has been recommended (Atkinson et al. 2005).

## Summary

When tested collaterally using a range of submaximal steady-state cycle ergometer tests, the duplicate ParvoMedics TrueOne 2400 systems produced total within-subject variations for *V*O_2_, *V*CO_2_ and *V*_E_ that each equated to ~4 % CV and ~6 % APE. The simultaneous tests demonstrated that the inter-unit technological error equated to ~1.5 % CV and ~2.1 % APE, thus the biological error within the collateral tests amounted to ~3.5 % CV and ~4 % APE. The few statistically significant differences detected between the duplicate systems were considered to have small or trivial effect sizes and their magnitudes to be of little physiological importance. Overall, the two ParvoMedics systems demonstrated very high inter-unit agreement and exceeded the performance criteria often expected of gas analysis systems.

## Abbreviations

APE: Absolute percentage error
CO_2_: Carbon dioxide
CV: Coefficient of variation
*F*_ECO2_: Fraction of expired carbon dioxide
*F*_EO2_: Fraction of expired oxygen
L min^−1^: Litres per minute
LOA: Limits of agreement
N_2_: Nitrogen
O_2_: Oxygen
RER: Respiratory exchange ratio
*V*O_2_: Oxygen consumption
*V*O_2max_: Maximal oxygen consumption
*V*CO_2_: Carbon dioxide production
*V*_E_: Expired ventilation
*V*_Emax_: Maximal expired ventilation
W: Watts

## References

[CR1] Armstrong LE, Costill DL (1985). Variability of respiration and metabolism: responses to submaximal cycling and running. Res Q Exerc Sport.

[CR2] Atkinson G, Davison RC, Nevill AM (2005). Performance characteristics of gas analysis systems: what we know and what we need to know. Int J Sports Med.

[CR3] Atkinson G, Nevill AM (1998). Statistical methods for assessing measurement error (reliability) in variables relevant to sports medicine. Sports Med.

[CR4] Babineau C, Leger L, Long A, Bosquet L (1999). Variability of maximum oxygen consumption measurement in various metabolic systems. J Strength Cond Res.

[CR5] Bassett DR, Howley ET, Thompson DL, King GA, Strath SJ, McLaughlin JE, Parr BB (2001). Validity of inspiratory and expiratory methods of measuring gas exchange with a computerized system. J Appl Physiol.

[CR6] Becque MD, Katch V, Marks C, Dyer R (1993). Reliability and within subject variability of VE, *V*O_2_, heart rate and blood pressure during submaximum cycle ergometry. Int J Sports Med.

[CR7] Bingisser R, Kaplan V, Scherer T, Russi EW, Bloch KE (1997). Effect of training on repeatability of cardiopulmonary exercise performance in normal men and women. Med Sci Sports Exerc.

[CR8] Bland JM, Altman DG (1986) Statistical methods for assessing agreement between two methods of clinical measurement. Lancet i:307–310 2868172

[CR9] Carter J, Jeukendrup AE (2002). Validity and reliability of three commercially available breath-by-breath respiratory systems. Eur J Appl Physiol.

[CR10] Clark NA, Edwards AM, Morton RH, Butterly RJ (2008). Season-to-season variations of physiological fitness within a squad of professional male soccer players. J Sport Sci Med.

[CR11] Cohen J (1988). Statistical power analysis for the behavioral sciences.

[CR12] Cooper JA, Watras AC, O’Brien MJ, Luke A, Dobratz JR, Earthman CP, Schoeller DA (2009). Assessing validity and reliability of resting metabolic rate in six gas analysis systems. J Am Diet Assoc.

[CR13] Crouter SE, Antczak A, Hudak JR, DellaValle DM, Haas JD (2006). Accuracy and reliability of the ParvoMedics TrueOne 2400 and MedGraphics VO2000 metabolic systems. Eur J Appl Physiol.

[CR14] Douglas CG (1911) A method for determining the total respiratory exchange in man. J Physiol 42:17–18

[CR15] Froelicher VF, Brammell H, Davis G, Noguera I, Stewart A (1974). A comparison of the reproducibility and physiologic response to three maximal treadmill exercise protocols. Chest.

[CR16] Gagnon J, Province MA, Bouchard C, Leon AS, Skinner JS, Wilmore JH, Rao DC (1996). The HERITAGE Family Study: quality assurance and quality control. Ann Epidemiol.

[CR17] Gore CJ, Catcheside PG, French SN, Bennett JM, Laforgia J (1997). Automated *V*O_2max_ calibrator for open-circuit indirect calorimetry systems. Med Sci Sports Exerc.

[CR18] Gore CJ, Gore CJ (2000). Quality assurance in exercise physiology laboratories. Physiological testing for elite athletes (Australian Sports Commission).

[CR19] Hodges LD, Brodie DA, Bromley PD (2005). Validity and reliability of selected commercially available metabolic analyzer systems. Scand J Med Sci Sports.

[CR20] Holm S (1979). A simple sequentially rejective multiple test procedure. Scand J Stat.

[CR21] Howley ET, Bassett DR, Welch HG (1995). Criteria for maximal oxygen uptake: review and commentary. Med Sci Sports Exerc.

[CR22] James DVB, Doust JH (1997). Reliability of pulmonary *V*O_2_ measurement and implications for determination of recovery from running. J Sports Sci.

[CR23] Jones NL (1984). Evaluation of a microprocessor-controlled exercise testing system. J Appl Physiol.

[CR24] Jones NL, Kane JW (1979). Quality control of exercise test measurement. Med Sci Sports Exerc.

[CR25] Katch VL, Sady SS, Freedson P (1982). Biological variability in maximum aerobic power. Med Sci Sports Exerc.

[CR26] Macfarlane DJ (2001). Automated metabolic gas analysis systems: a review. Sports Med.

[CR27] Macfarlane DJ, Wong P (2011). Validity, reliability and stability of the portable Cortex Metamax 3B gas analysis system. Eur J Appl Physiol.

[CR28] McArdle WD, Katch FI, Pechar GS (1973). Comparison of continuous and discontinuous treadmill and bicycle tests for max *V*O_2_. Med Sci Sports Exerc.

[CR29] McClain JJ, Sisson SB, Tudor-Locke C (2007). Actigraph accelerometer interinstrument reliability during free-living in adults. Med Sci Sports Exerc.

[CR30] Miles DS, Cox MH, Verde TJ (1994). Four commonly utilized metabolic systems fail to produce similar results. Sports medicine, training and rehabilitation.

[CR31] Mitchell JH, Sproule BJ, Chapman CB (1958). The physiological meaning of the maximal oxygen intake test. J Clin Invest.

[CR32] Nordrehaug JE, Danielson R, Stangeland L, Rosland GA, Viko-Mo H (1991). Respiratory gas exchange during treadmill exercise testing: reproducibility and comparison of different exercise protocols. Scand J Clin Lab Invest.

[CR33] Rosdahl H, Gullstrand L, Salier-Eriksson J, Johansson P, Schantz P (2009). Evaluation of the Oxycon Mobile metabolic system against the Douglas bag method. Eur J Appl Physiol.

[CR34] Shephard RJ, Rankinen T, Bouchard C (2004). Test-retest errors and the apparent heterogeneity of training response. Eur J Appl Physiol.

[CR35] Skinner JS, Wilmore KM, Jaskolska A, Jaskolski A, Daw EW, Rice T, Gagnon J, Leon AS, Wilmore JH, Rao DC, Bouchard C (1999). Reproducibility of maximal exercise test data in the HERITAGE Family Study. Med Sci Sports Exerc.

[CR36] Taylor C (1944). Some properties of maximal and submaximal exercise with reference to physiological variation and the measurement of exercise tolerance. Am J Physiol.

[CR37] Taylor HL, Buskirk E, Henschel A (1955). Maximal oxygen uptake as an objective measure of cardio-respiratory performance. J Appl Physiol.

[CR38] Vogler AJ, Rice AJ, Gore CJ (2010). Validity and reliability of the Cortex MetaMax3B portable metabolic system. J Sports Sci.

[CR39] Wilmore JH, Standorth PR, Turley KR, Gagnon J, Daw EW, Leion AS, Rao DC, Skinner JS, Bouchard C (1998). Reproducibility of cardiovascular, respiratory, and metabolic responses to submaximal exercise: the HERITAGE Family Study. Med Sci Sports Exerc.

[CR40] Withers RT, Gore CJ, Gass G, Hahn A, Gore CJ (2000). Determination of maximal oxygen consumption (*V*O_2_max) or maximal aerobic power. Physiological testing for elite athletes (Australian Sports Commission).

[CR41] Yule E, Kaminsky LA, Sedlock DA, King BA, Whaley MH (1996) Inter-laboratory reliability of *V*O_2max_ and submaximal measurements. Med Sci Sports Exerc 28:S15 #87

